# Bilateral large inguinal hernia with buried penis–a rare case report

**Published:** 2011-05-25

**Authors:** R Singal, A Gupta, S Goyal, B Singh, S Gupta

**Affiliations:** * Department of Surgery–Maharishi Markandeshwer Institute of Medical Sciences and Research, Mullana, (Distt–Ambala), Haryana India; ** Department of Anatomy, Adesh Institute of Medical Sciences and Research, Bathinda, PunjabIndia; *** Department of Radiodiagnosis, Maharishi Markandeshwer Institute of Medical Sciences and Research, Mullana (Distt–Ambala) Pin Code–133201, HaryanaIndia

## Case presentation

A 7–year–old child reported with swelling in the bilateral inguinoscrotal region. Parents have noticed swelling just after the 6 months from birth. There was also a complaint of penile shortening as far as its size is concerned. No other complaints were present.

**Figure 1:**
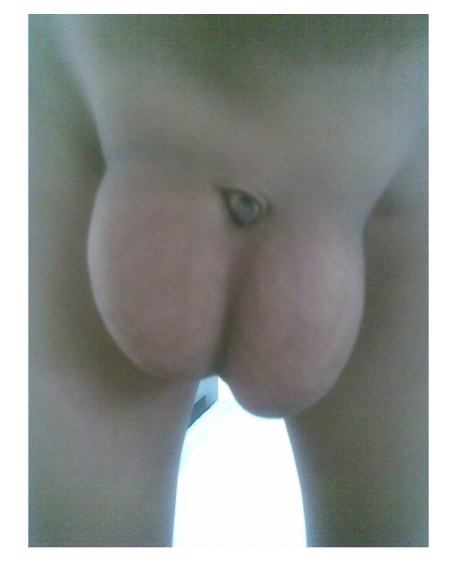
Large bilateral inguinal hernia and buried penis

The vitals were stable. On local examination, bilateral inguino–scrotal swelling was present of a size about 8 x 6 cm on both sides, firm in consistency, non–tender and the skin was normal in appearance (Figure 1).

**Figure 2:**
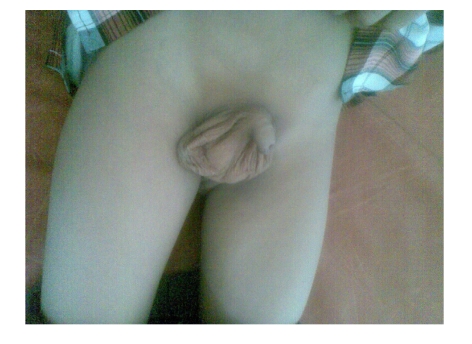
After the reduction of the hernia the penis was normal

Swelling was reducible on lying down and cough impulse was present (Figure 2).

The penis was buried inside and the testis was felt separately.

The incision was made over the skin crease, opened in layers and the cord was identified. Then sac was identified and separated from the cord. Contents were small bowel loops, which were reduced and the sac was transfixed with Vicryl 3–0.  Herniotomy was done. The same procedure was done to the other side. The child was discharged in a satisfactory condition. The child is doing well in the follow up period of 6 months.

Inguinal hernia is one of the most common pediatric surgical problems and when treated early and appropriately it is associated with negligible morbidity and very rarely to any mortality [1].The incidence of hernias is of 10–20 per 1000 live births and it is much more common following premature birth. While hernia location is more common on the right side, as many as 10% are bilateral. Inguinal hernias continue to be the most common congenital pathology in children needing surgical repair early in life. 

Approximately 1–3% of the children have an inguinal hernia. The incidence is higher in premature babies (3–5%). Almost all inguinal hernias in children are of indirect type (99%). The few direct hernias in children are the result of previous surgery or inguinal floor disruption [2]. Inguinal hernia in children and young adults is usually the result of patent processes vaginalis. It can be life threatening, or can lead to the loss of the testis or part of the intestine if strangulation occurs. Incidence of inguinal hernia ranges between 0.8% and 4.4% [3, 4]
